# An unusual case of three concomitant primary solid cancers with unique histopathological characteristics

**DOI:** 10.1016/j.ijscr.2023.109080

**Published:** 2023-11-20

**Authors:** Mohamed Shatila, Ijaz Ahmad, Janos Balega, Maninder S. Kalkat, Akshay J. Patel

**Affiliations:** a Department of Thoracic Surgery, University Hospitals Birmingham NHS Trust, UK; b Department of Cardiothoracic Surgery, Faculty of Medicine, Alexandria University Hospital, Alexandria, Egypt; c Department of Head and Neck Surgery, University Hospitals Birmingham NHS Trust, UK; d Department of Gynaecological Oncology, City Hospital, Birmingham, UK; e Institute of Immunology and Immunotherapy, University of Birmingham, UK

**Keywords:** Ovarian teratoma, Struma ovarii (SO), Papillary thyroid carcinoma (PTC), Lung adenocarcinoma, Non-small cell lung cancer (NSCLC), Radioactive iodine (RAI)

## Abstract

**Introduction and importance:**

Struma Ovarii is a rare type of monodermal teratoma with at least 50 % of its mass being thyroid tissue. They make up <1 % of all ovarian tumours and 3 to 5 % of all ovarian teratomas. These tumours are usually benign but malignant transformation is seen in <5 % of cases.

**Case presentation:**

We present the case of a 45-year-old lady with three synchronous primary cancers on a background of Struma Ovarii; primary lung adenocarcinoma, papillary thyroid carcinoma and ovarian teratoma. Over the course of 18 months, this lady underwent full pelvic clearance of malignant Struma Ovarii and lymph nodes, total thyroidectomy, and an anatomical lung resection.

**Clinical discussion:**

This case represents an incredibly rare condition of Struma Ovarii for which there is no firm management consensus. Furthermore, the uniqueness of three separate primaries has to the best of our knowledge not previously been reported in the literature.

**Conclusion:**

This reinforces the notion that in select patients, radical management with curative intent is entirely possible but requires complete multi-disciplinary and multi-modal sub-specialty collaboration.

## Highlights

• This case represents a rare condition of Struma Ovarii for which there is no firm management consensus.
• SO is a rare type of monodermal teratoma with at least 50 % of its mass being thyroid tissue.
• The combination of these three separate entities is unique and not described in somebody without risk factors.
• A non-smoker, a person of young age and no oncogenic drivers suggesting cause for somatic mutational drivers as opposed to germline mutations.

## 1 Introduction

Ovarian teratoma, a histological subtype of ovarian germ cell tumour, is divided into immature, mature, monodermal and mixed types [1]. The monodermal teratoma is further divided into Struma Ovarii, Carcinoid, Struma Ovarii (SO) and Carcinoid and others (e.g., malignant neuroectodermal and ependymoma) [2].

SO is a rare type of monodermal teratoma with at least 50 % of its mass being thyroid tissue [3]. They make up <1 % of all ovarian tumours and 3 to 5 % of all ovarian teratomas [4]. These tumours are usually benign but malignant transformation is seen in <5 % of cases [5]. The common malignant forms are papillary and follicular carcinoma [6]. They are more likely to occur in the age group from 40 to 60 years although they could occur in all age groups [7].

Here we discuss a 45-year-old female presented with a non-invasive well differentiated papillary thyroid carcinoma on a background of SO, with a contralateral ovarian teratoma, papillary thyroid carcinoma and an incidental finding of a left upper lobe pathologically staged T1bN0M0 adenocarcinoma of the lung.

## 2 Case presentation

**Fig. 1:**
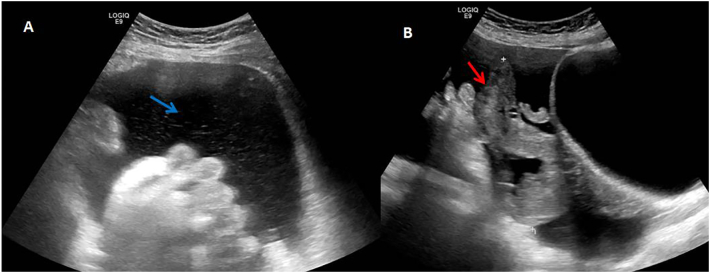
Pelvic ultrasound scans demonstrating moderate volume ascites (panel A, left hand side, blue arrow) and a significant 11 cm in maximal length adnexal mass.

A 45-year-old non-smoking lady with a performance status of zero and no known co-morbidities or significant family history presented with lower abdominal symptoms of bloating, left sided mass and right groin discomfort. The patient was reviewed at the gynaecology outpatient clinic and after clinical examination she was referred for transabdominal and transvaginal pelvic ultrasound. This revealed moderate volume ascites (Fig. 1A – blue arrow) with a complex irregular cystic/solid mass in the pelvis measuring approximately 10.5*4.9*8.7 cm (Fig. 1B – red arrow). Findings were concerning of an underlying ovarian malignancy and an urgent CT scan, and CA-125 level were advised. CA-125 levels were elevated (744 U/ml), and the case was subsequently discussed in the Gynaecology MDT.

The consensus was to proceed with a diagnostic laparoscopy. This revealed a large pelvic mass with large amount of fluid but with no evidence of cancer spread and hence conversion to midline laparotomy was made. A large 11 cm left adnexal mass was seen with signs of long-term rupture and fluid pouring out of a presumed cyst. A 1 cm deposit was noted on the Pouch of Douglas (POD) on the right uterosacral ligament. Intraoperative impression was that of a stage 2 borderline ovarian tumour (BOT) owing to the peritoneal nodules seen in the POD with preoperative cystic rupture. Pelvic clearance with total omentectomy was performed and all tissue sent for pathology.

Pathological examination of the left adnexal mass revealed an 11*9.3*5.8 cm mass with a slightly nodular smooth shiny surface with an area of discontinuity. Cut surface was solid and polycystic. The cystic areas contained mucoid material with pale tan and slightly haemorrhagic tissue in the solid component. Microscopic examination revealed admixed thyroid like follicles that contained colloid and were CD56, AE1/3 and TTF1 positive. The features were suggestive of SO and further opinion was needed from a head and neck pathologist. Other findings were superficial adenomyosis, mature cystic teratoma of the right ovary and the POD showed haemorrhagic granulation tissue secondary to endometriosis. The omentum was tumour free and ascitic fluid cytology showed reactive appearance with no features of malignancy. Complete resection of the disease was achieved.

**Fig. 2:**
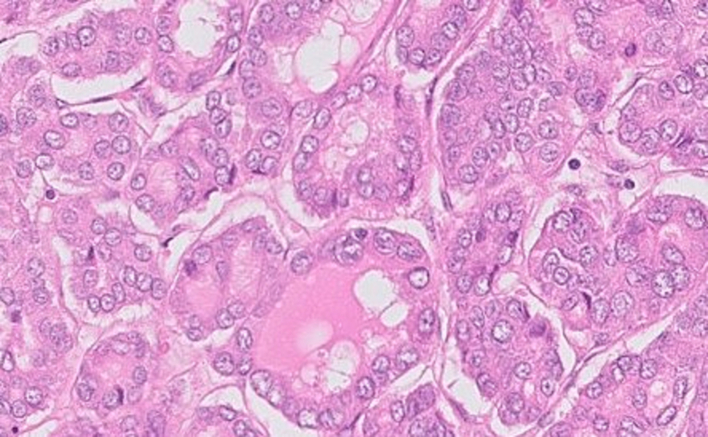
H&E pathology slide demonstrating malignant struma ovarii.

Further pathology opinion best regarded the diagnosis as primary non-invasive well differentiated papillary thyroid carcinoma, ex monodermal ovarian teratoma (Struma Ovarii) (Fig. 2). There was no evidence of lympho-vascular invasion or extra-ovarian spread. Thyroid transcription factor 1, CD56, broad spectrum cytokeratins (AEVAE3) and thyroglobulin immunostaining reactivity gave support to the interpretation. Advice was given to exclude metastatic disease from elsewhere and to seek potential secondary disease outside the pelvis.

**Fig. 3:**
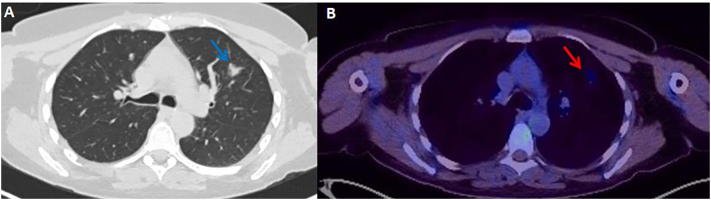
PET-CT demonstrates a 16 mm spiculate solid mass in the left upper lobe (panel A, left hand side, blue) on CT reconstruction. The PET component (panel B, right hand side, red arrow) shows the same mass taking up low levels of FDG tracer.

Follow up PET-CT scan revealed a small, partially calcified thyroid nodule within the upper pole of the right lobe, with faint uptake and an SUV max of 3.2. A 16 mm part solid left upper lobe lung nodule with faint uptake was also seen with an SUV max of 2.4 (Fig. 3A and B). A border line enlarged pelvic lymph node was seen with no FDG uptake.

**Fig. 4:**
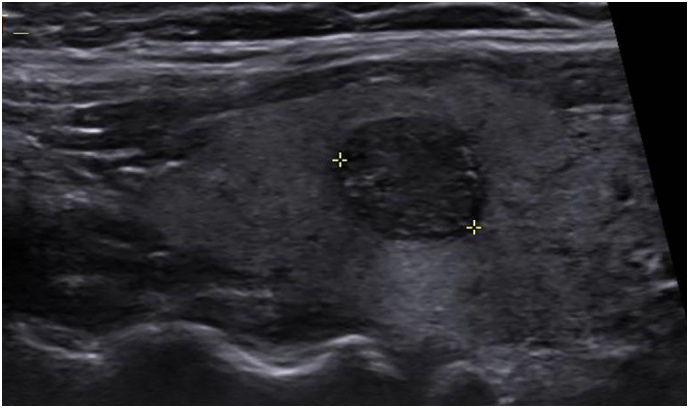
Ultrasound of the neck demonstrates a solid mass in the right lobe of the thyroid, demarcated between the two yellow cross hairs.

The patient went to have an ultrasound of the neck to assess for evidence of underlying thyroid carcinoma. This revealed a well-defined solid nodule measuring 8*8.5*9 mm with well-defined borders in the right lobe of the thyroid, with appearances suspicious for a papillary thyroid carcinoma (Fig. 4). There was no local extra thyroid extension within the neck or capsular breach. No enlarged lymph nodes were seen. Fine Needle Aspiration (FNA) of the right thyroid nodule was done which revealed features suggestive of papillary thyroid carcinoma (Thy5 positive). Routine haematology and thyroid function tests at this stage were otherwise normal however serum thyroglobulin was elevated. BRAF and RET/PTC rearrangements were negative. Results were discussed in the thyroid MDT and the patient went on to have a total thyroidectomy however elected not to undergo further treatment with Radioactive Iodine (RAI) therapy.

Following the patient's thyroid surgery, a follow up scan was requested to assess for the left upper lobe pulmonary nodule. Results were discussed with the lung MDT and the patient had a CT guided biopsy which showed adenocarcinoma of lung origin. The lesion underwent molecular testing, and this revealed no oncogenic driver mutations (EGFR/KRAS/BRAF/ALK/ROS/NTRK negative, PDL1 < 1 %). Patient subsequently underwent a Video-Assisted Thoracoscopic (VAT) left upper lobectomy, pathologically staged as a pT1bpN0 adenocarcinoma. No adjuvant treatment was administered.

The patient recovered well post all these interventions. Given the toll of the surgeries and the psychological impact of three concurrent types of cancer, the patient underwent counselling which helped a great deal and has contributed to a long-lasting recovery.

## 3 Discussion

The average female age of diagnosis with SO is 43, similar to our case [8]. The lack of sufficient literature and rarity of the disease have made the diagnosis of SO to be difficult with no robust or standardised diagnostic or management criteria. Presenting symptoms include pelvic pain, abdominal mass, and discomfort, all seen in our case. Moderate ascites on CT scan have been seen in patients with SO, although in our case it was due to cyst rupture. >90 % of patients are euythyroid with an elevated CA-125 level. Elevated CA-125 levels have shown to be of little benefit in aiding in the diagnosis of SO because high levels are seen in both benign and malignant tumours of the ovary [9]. The diagnosis of SO can only be made after histopathological analysis [10].

In the medical literature, <17 patients were reported with Malignant SO and coexisting thyroid carcinoma [11]. Only one case has presented with follicular carcinoma in the SO and papillary carcinoma in the thyroid, all other patients had papillary thyroid carcinoma in both sites, similar to our case [12]. Ovarian mass in most of the cases were >5 cm with the thyroid mass ranging from 0.2 to 1.7 cm [11].

It is Important to differentiate between 2 synchronous primaries and thyroid metastasis. The presence of thyroid epithelial tissues and teratomatous elements favours the diagnosis of two primaries [13]. The use of ovarian MRI can also help in distinguishing between the two possibilities. A multiloculated ovarian cystic mass with solid component and variable signal intensity between the locules favours 2 separate primaries [14].

The rarity of the disease led to a lot of controversy in management, especially when the patient's fertility is an issue [[15], [16], [17]]. When the preservation of fertility is not needed and/or there is evidence of tumour metastasis, an aggressive approach is recommended including Total Abdominal Hysterectomy with Bilateral Salpingo-oophorectomy (TAH-BSO), lymph node clearance and omentectomy, the approach that was performed in our patient [18].

Thyroidectomy and Radioactive Iodine is indicated in patients with metastasis, but it is a point of debate in non-metastatic malignant SO [19]. In patients with a synchronous primary thyroid cancer, total thyroidectomy with radioactive iodine (RAI) reported no recurrence compared to a 21 % rate of recurrence in patients who had total thyroidectomy alone [[19], [20]].

What makes our case even rarer is the incidental finding of a left upper lobe mass of the lung. CT guided biopsy had confirmed the evidence of adenocarcinoma. PET scan excluded mediastinal and hilar lymph node spread and the patient had a left video assisted thoracoscopic upper lobectomy with a smooth postoperative recovery. With a pathological staging of T1bN0, our patient will only be required to do serial follow up CT scans of the thorax and upper abdomen for 5 years as part of her surgical surveillance. As far as literature is concerned, no previous report of 3 similar primaries have been published. At most recent follow-up, the patient is radiologically disease-free, maintaining normal haematological, endocrine, and thyroid function parameters. This work has been reported in line with the SCARE criteria [21].

## 4 Conclusions

The combination of these three separate entities is indeed unique and not already described particularly in somebody without significant risk factors, a non-smoker, a person of young age and no major oncogenic drivers suggesting high cause for somatic mutational drivers as opposed to germline mutations which in itself is of unique interest.

## Consent for publication

Consent was sought from the patient in question.

## Ethical approval

There was no ethical approval needed as this did not directly involve patients.

## Funding

There are no sources of funding to declare.

## Author contribution

All authors contributed to this piece of work. Authors (MS, AJP) designed the study, gathered the data, and wrote the manuscript. Other authors (IA, JB, MSK, AJP) provided analytical support and supervised writing of the manuscript.

## Guarantor

AJP is the guarantor of this work.

## Research registration number

None to declare.

## Declaration of competing interest

There are no known conflicts of interest.

## Data Availability

All data and materials are available upon reasonable request from the corresponding author.

## References

[1] Kim D., Cho H.C., Park J.W., Lee W.A., Kim Y.M., Chung P.S. (2009). Struma ovarii and peritoneal strumosis with thyrotoxicosis. Thyroid.

[2] Gershenson D.M., Morris M., Cangir A. (1990). Treatment of malignanat germ cell tumors of the ovary with bleomycin, etoposide, and cisplatin. Clin. Oncol..

[3] Botros K., Noor Chelsea N., Bermingham J. (2021). Struma ovarii: a thyroxine-producing ovarian tumor in pregnancy. Cureus.

[4] Podfigurna A., Szeliga A., Horwat P., Maciejewska-Jeske M., Meczekalski B. (2021). Gynecol. Endocrinol..

[5] Mcgill J., Sturgeon C., Angelos P. (2009). Metastatic struma ovarii treated with total thyroidectomy and radioiodine ablation. Endocr. Pract..

[6] Yassa L., Sadow P., Marqusee E. (2008). Malignant struma ovarii. Nat. Clin. Pract. Endocrinol. Metab..

[7] Goffredo P., Sawka A.M., Pura J., Adam M.A., Roman S.A., Sosa J.A. (2015). Malignant struma ovarii: a population-level analysis of a large series of 68 patients. Thyroid.

[8] Zhu Y., Wang C., Zhang G.N., Shi Y., Xu S.Q., Jia S.J., He R. (2016). Papillary thyroid cancer located in malignant struma ovarii with omentum metastasis: a case report and review of the literature. World J. Surg. Oncol..

[9] Szczepanek-Parulska E., Pioch A., Cyranska-Chyrek E. (2019). The role of immunohistochemical examination in diagnosis of papillary thyroid cancer in struma ovarii. Folia Histochem. Cytobiol..

[10] Zhang T., Chen P., Gao Y. (2018). Struma ovarii: a mini review. Int. J. Clin. Exp. Med..

[11] Elias G. Tzelepis, Elena Barengolts, Steven Garzon, Joseph Shulan, and Yuval Eisenberg, “Unusual Case of Malignant Struma Ovarii and Cervical Thyroid Cancer Preceded by Ovarian Teratoma: Case Report and Review of the Literature Elias G. Tzelepis. 10.1155/2019/7964126 PMC6441504 31007958

[12] Janszen E.W., Van Doorn H.C., Ewing P.C. (2008). Malignant struma ovarii: good response after thyroidectomy and I ablation therapy. Clin. Med. Oncol..

[13] Leong A., Roche P.J., Paliouras M., Rochon L., Trifiro M., Tamilia M. (2013). Coexistence of malignant struma ovarii and cervical papillary thyroid carcinoma. J. Clin. Endocrinol. Metab..

[14] Leite I.T., Cunha T.M., Figueiredo J.P., Felix A. (2013). Papillary carcinoma arising in struma ovarii versus ovarian metastasis from primary thyroid carcinoma: a case report and review of the literature. J. Radiol. Case Rep..

[15] Gunasekaran S., Kopecka E., Maung K.H., England R.J. (2012). Struma ovarii and the thyroid surgeon. J. Laryngol. Otol..

[16] Luo J., Xie C., Li Z. (2014). Treatment for malignant struma ovarii in the eyes of thyroid surgeons: a case report and study of Chinese cases reported in the literature. Medicine.

[17] DeSimone C.P., Lele S.M., Modesitt S.C. (2003). Malignant struma ovarii: a case report and analysis of cases reported in the literature with focus on survival and I131 therapy. Gynecol. Oncol..

[18] Goffredo P., Sawka A.M., Pura J., Adam M.A., Roman S.A., Sosa J.A. (2015). Malignant struma ovarii: a population-level analysis of a large series of 68 patients. Thyroid.

[19] DeSimone C.P., Lele S.M., Modesitt S.C. (2003). Malignant struma ovarii: a case report and analysis of cases reported in the literature with focus on survival and I131 therapy. Gynecol. Oncol..

[20] Jean S., Tanyi J.L., Montone K., McGrath C., Lage- Alvarez M.M., Chu C.S. (2012). Papillary thyroid cancer arising in struma ovarii. J. Obstet. Gynaecol..

[21] Agha R.A., Franchi T., Sohrab C., Mathew G., Kirwan A., Thomas A. (2020). The SCARE 2020 guideline: updating consensus Surgical Case Report (SCARE) guidelines. Int. J. Surg..

